# New Species of *Nectriaceae* (*Hypocreales*) from China

**DOI:** 10.3390/jof8101075

**Published:** 2022-10-13

**Authors:** Zhao-Qing Zeng, Wen-Ying Zhuang

**Affiliations:** State Key Laboratory of Mycology, Institute of Microbiology, Chinese Academy of Sciences, Beijing 100101, China

**Keywords:** *Ascomycota*, morphology, multigene analyses, taxonomy

## Abstract

Species of *Nectriaceae* commonly occur on living and decaying woody substrates, soil, fruitbodies of other fungi, and insects. Some of them are reported as endophytes, opportunistic pathogens of crops and humans, or producers of mycotoxins. To explore the species diversity of the family, specimens from different regions of China were collected and examined. Four novel taxa of *Penicillifer*, *Pseudocosmospora*, and *Thelonectria* were introduced on the basis of morphological characteristics and DNA sequence analyses of combined datasets of the act, ITS, LSU, rpb1, rpb2, tef1, and tub2 regions. Differences between the new species and their close relatives were compared and discussed.

## 1. Introduction

The family *Nectriaceae* was established in 1865 to accommodate those fungi producing uniloculate perithecia that are yellow, orange-red to purple, or brown; often change color in 3% potassium hydroxide (KOH) and 100% lactic acid (LA); and have a tropical and subtropical distribution [1]. Approximately 69 genera are currently accepted [1,2,3], including genera such as *Penicillifer* Emden, *Pseudocosmospora* C.S. Herrera & P. Chaverri, and *Thelonectria* P. Chaverri & C. Salgado. The generic concepts and phylogenetic relationships of the family were comprehensively stated by Lombard et al. [2].

The genus *Penicillifer*, typified by *Penicillifer pulcher* Emden., was introduced by Emden [4]. It was treated as the asexual stage of *Viridispora* Samuels & Rossman [1] and recommended as the correct name for this group of fungi [2]. Seven species are currently known in the genus [1,2,5]. The genus *Pseudocosmospora*, typified by *P*. *eutypellae* C.S. Herrera & P. Chaverri, was established by Herrera et al. [6] to accommodate *Cosmospora vilior* (Starbäck) Rossman & Samuels and related species that are usually fungicolous. Sixteen species are recognized [6,7,8,9]. The genus *Thelonectria*, typified by *T*. *discophora* (Mont.) P. Chaverri & C. Salgado, was established by Chaverri et al. [10] to include the species formerly placed in the *Nectria mammoidea* and *N. veuillotiana* groups with a cosmopolitan distribution [11]. Forty-seven species are accepted in the genus [10,11,12,13,14,15,16,17,18].

In our study of the hypocrealean specimens from different regions of China, four unusual fungi were encountered. Judging by their perithecial gross morphology, anatomy, and culture characteristics, they represent four undescribed species of *Penicillifer*, *Pseudocosmospora*, and *Thelonectria*. Their taxonomic placements were further confirmed by multigene phylogenetic analyses of α-actin (act), nuclear ribosomal DNA ITS1-5.8S-ITS2 (ITS), large subunit of nuclear ribosomal DNA (LSU), the largest subunit of RNA polymerase II (rpb1), the second largest subunit of RNA polymerase II (rpb2), translation elongation factor 1-α (tef1), and β-tubulin (tub2). The differences between the novel taxa and their close relatives were compared.

## 2. Materials and Methods

### 2.1. Sampling and Morphological Studies

Specimens were collected from Beijing and the Guangxi Zhuang Autonomous Region, and they are preserved in Herbarium Mycologicum Academiae Sinicae (HMAS). Cultures were obtained by single ascospore isolation from the fresh perithecium and deposited in the China General Microbiological Culture Collection Center (CGMCC). The method of Lombard et al. [2] was followed for morphological observations. The ascomatal wall reactions to 3% KOH and 100% LA were tested. Sections were prepared with a freezing microtome (YD-1508-III, Jinhua, China) at a thickness of 6–8 μm for anatomic examination. Lactophenol cotton blue solution was used as a mounting medium for the measurements of the perithecia, asci, ascospores, conidiophores, and conidia. Photographs were taken with a Leica DFC450 digital camera (Wetzlar, Germany) attached to a Leica M125 stereomicroscope (Milton Keynes, UK) for gross morphology and a Zeiss AxioCam MRc 5 digital camera (Jena, Germany) attached to a Zeiss Axio Imager A2 microscope (Göttingen, Germany) for microscopic features. For colony morphology and growth rates, strains were grown on potato dextrose agar (PDA, 20% *w/v* potato + 2% *w/v* dextrose + 2% *w/v* agar) and synthetic nutrient-poor agar (SNA) [19] in 90 mm plastic Petri dishes at 25 °C for 14 d with alternating periods of light and darkness (12 h/12 h).

### 2.2. DNA Extraction, PCR Amplification, Sequencing, and Phylogenetic Analyses

Genomic DNA was extracted from fresh mycelium following the method of Lombard et al. [2]. Seven primer pairs, act1/act2 [20], ITS5/ITS4 [21], LR0R/LR5 [22,23], rpb1a/rpb1c [24], RPB2-5f/RPB2-7cR [25], 728F/EF2 [26,27], and T1/T22 [28], were used to amplify the sequences of the act, ITS, LSU, rpb1, rpb2, tef1, and tub2 regions, respectively. PCR reactions were performed using an ABI 2720 Thermal Cycler (Applied Biosciences, Foster City, CA, USA), and DNA sequencing was carried out in both directions on an ABI 3730XL DNA Sequencer (Applied Biosciences, Foster City, CA, USA).

Newly obtained sequences and those retrieved from GenBank are listed in Table 1, Table 2 and Table 3. The sequences were assembled and aligned, and the primer sequences were trimmed using BioEdit 7.0.5 [29] and converted to nexus files by ClustalX 1.83 [30]. A partition homogeneity test (PHT) was performed with 1000 replicates in PAUP*4.0b10 [31] to evaluate statistical congruence amongst these loci. The aligned sequences were combined in BioEdit and analyzed with Bayesian inference (BI), maximum likelihood (ML), and maximum parsimony (MP) methods to determine the phylogenetic positions of the new species. The BI analysis was conducted by MrBayes 3.1.2 [32] using a Markov chain Monte Carlo algorithm. Nucleotide substitution models were determined by MrModeltest 2.3 [33]. Four Markov chains were run simultaneously for 1,000,000 generations, with the trees sampled every 100 generations. A 50% majority rule consensus tree was computed after excluding the first 2500 trees as ‘burn-in’. The Bayesian inference posterior probability (BIPP) was determined from the remaining trees. Branch support measures were calculated with 1000 bootstrap replicates. The ML analysis was performed via IQ-Tree 1.6.12 [34] using the best model for each locus chosen by ModelFinder [35]. The MP analysis was performed with PAUP 4.0b10 [31] using heuristic searches with 1000 replicates of random addition of sequences and subsequent TBR (tree bisection and reconnection) branch swapping. The topological confidence of the resulting trees and the statistical supports of the branches were tested by maximum parsimony bootstrap proportion (MPBP) with 1000 replications, each with 10 replicates of random addition of taxa. Trees were examined by TreeView 1.6.6 [36]. Maximum likelihood bootstrap proportion (MLBP) and MPBP greater than 70% and BIPP greater than 90% were shown at the nodes.

## 3. Results

### 3.1. Phylogeny

**Figure 1:**
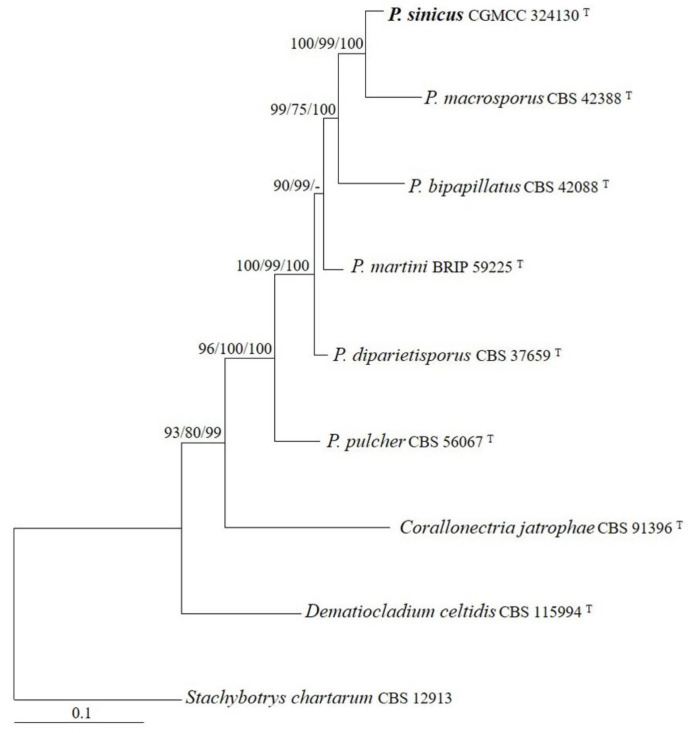
The maximum likelihood tree inferred from combined ITS, LSU, rpb2, and tef1 sequences of representative species of *Penicillifer*. MLBP (**left**) and MPBP (**middle**) values greater than 70% and BIPP (**right**) values greater than 90% are shown at the nodes.

The sequences of the ITS, LSU, rpb2, and tef1 regions from six *Penicillifer* species were analyzed. *Stachybotrys chartarum* (Ehrenb.) S. Hughes was used as the outgroup taxon. The partition homogeneity test (*p* = 0.01) indicated that the individual partitions were not highly incongruent [37]; thus, these four loci were combined for the phylogenetic analyses. The ML tree is shown in Figure 1. The topologies of the BI and MP trees were similar to that of the ML tree. The isolate CGMCC 3.24130 grouped with other members of *Penicillifer* and received high statistical support (MLBP/MLBP/BIPP = 96%/100%/100%).

**Figure 2:**
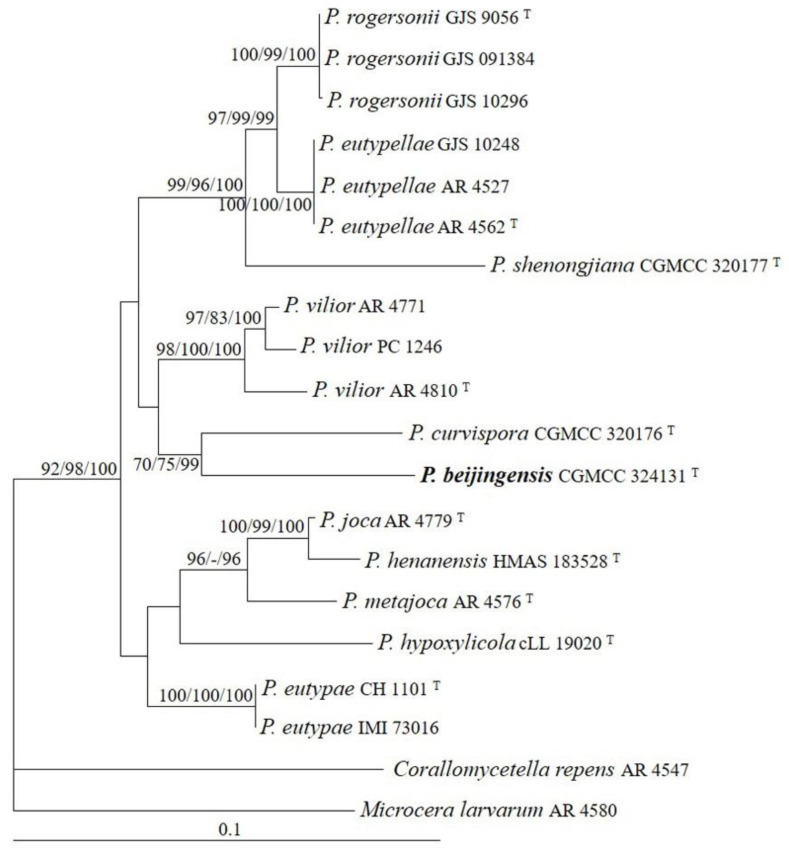
The maximum likelihood tree inferred from combined ITS, LSU, and tub2 sequences of representative species of *Pseudocosmospora*. MLBP (**left**) and MPBP (**middle**) values greater than 70% and BIPP (**right**) values greater than 90% were shown at the nodes.

The sequences of ITS, LSU, and tub2 regions from 11 *Pseudocosmospora* species were analyzed. *Corallomycetella repens* (Berk. & Broome) Rossman & Samuels and *Microcera larvarum* (Fuckel) Gräfenhan, Seifert & Schroers were used as outgroup taxa. The partition homogeneity test (*p* = 0.01) indicated that the individual partitions were not highly incongruent [37]; thus, these three loci were combined for the phylogenetic analyses. The ML tree is shown in Figure 2. The topologies of the BI and MP trees were similar to that of the ML tree. The isolate CGMCC 3.24131 grouped with other species of *Pseudocosmospora* and received high statistical support (MLBP/MLBP/BIPP = 92%/98%/100%).

**Figure 3:**
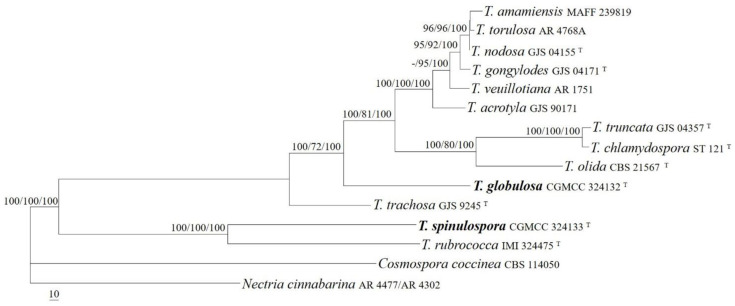
The maximum likelihood tree inferred from combined act, ITS, LSU, rpb1, and tub2 sequences of representative species of *Thelonectria*. MLBP (**left**) and MPBP (**middle**) values greater than 70% and BIPP (**right**) values greater than 90% were shown at the nodes.

The sequences of the act, ITS, LSU, rpb1, and tub2 regions from 13 *Thelonectria* species were analyzed. *Cosmospora coccinea* Rabenh. and *Nectria cinnabarina* (Tode) Fr. were used as outgroup taxa. The partition homogeneity test (*p* = 0.01) indicated that the individual partitions were not highly incongruent [37]; thus, these five loci were combined for the phylogenetic analyses. The ML tree is shown in Figure 3. The topologies of the BI and MP trees were similar to that of ML tree. The isolates CGMCC 3.24132 and CGMCC 3.24133 were well-located among other *Thelonectria* species and received high supporting values (MLBP/MLBP/BIPP = 100%/100%/100%). The isolate CGMCC 3.24133 was related to *T. rubrococca* (Brayford & Samuels) Salgado & P. Chaverri, receiving high statistic values (MLBP/MLBP/BIPP = 100%/100%/100%), and the strain CGMCC 3.24132 formed an independent lineage and was related to the *T. veuillotiana* complex (MLBP/MLBP/BIPP = 100%/72%/100%).

### 3.2. Taxonomy

**Figure 4:**
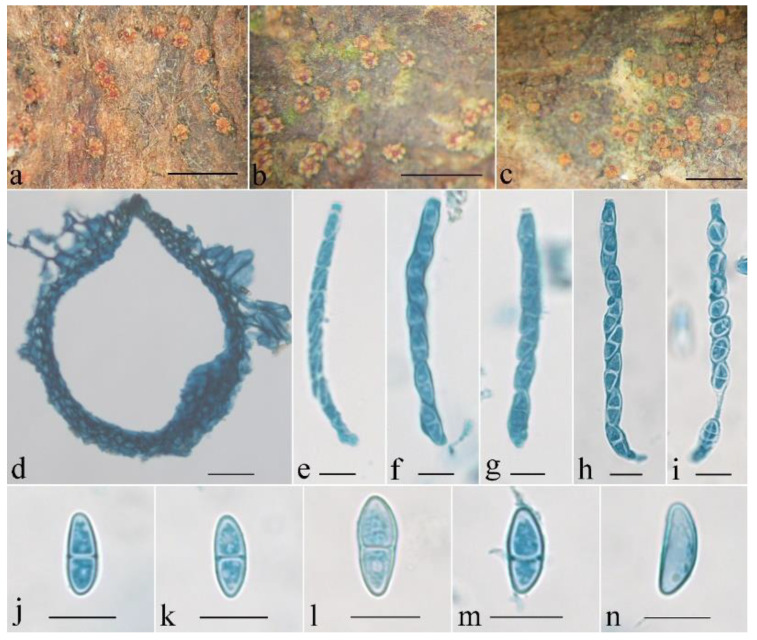
Macroscopic and microscopic morphology of *Penicillifer sinicus* (HMAS 247865). (**a**–**c**) Ascomata on natural substratum; (**d**) median section of perithecium in lactophenol cotton blue; (**e**–**i**) asci with ascospores in lactophenol cotton blue; (**j–n**) ascospore in lactophenol cotton blue. Bars: (**a**–**c**) = 1 mm; (**d**) = 50 μm; (**e**–**n**) = 10 μm.

**Figure 5:**
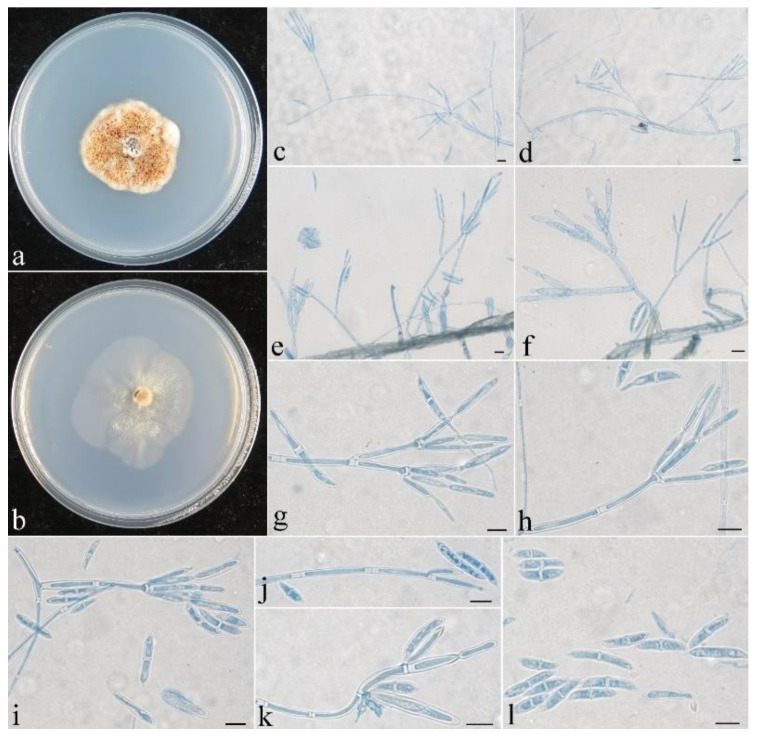
Colonial and microscopic morphology of *Penicillifer sinicus* (CGMCC 3.24130). (**a**) Colony after 1 week at 25 °C on PDA; (**b**) colony after 1 week at 25 °C on SNA; (**c**–**k**) conidiophores, phialides, and macroconidia in lactophenol cotton blue; (**l**) macroconidia in lactophenol cotton blue. Bars: (**c**–**l**) = 10 μm.

***Penicillifer sinicus*** Z.Q. Zeng & W.Y. Zhuang, sp. nov. Figure 4 and Figure 5.

**Fungal Names:** FN571297.

**Etymology:** The epithet refers to the country where the fungus was collected.

**Typification:** CHINA, Guangxi Zhuang Autonomous Region, Guilin City, Mao’er Mountain, on rotten twigs, 7 December 2019, Z.Q. Zeng & H.D. Zheng 12496 (holotype HMAS 247865, ex-type strain CGMCC 3.24130).

**GenBank accession numbers:** ITS OP223439, LSU OP223435, rpb2 OP272863, tef1 OP272864, rpb1 OP586759, tub2 OP586763.

Mycelium was not visible on the natural substratum. Perithecia were superficial, solitary, non-stromatic or with a basal stroma, and subglobose to globose with an acute to blunt papilla; the surface was warted; they did not collapse upon drying; they were yellowish brown to brown and did not change color in 3% KOH or 100% LA, and the size was 235–314 × 176–274 μm. The perithecial surface had warts 15–55 μm high. Perithecial walls were two-layered, 23–35 μm thick; the outer layer was of the textura angularis, 16–25 μm thick, with cell 5–9 × 4–8 μm, and cell walls 1–1.2 μm thick; the inner layer was of the textura prismatica, 7–10 μm thick, with cell 5–13 × 2–3 μm, and cell walls 0.8–1 μm thick. Asci were unitunicate, cylindrical, and eight-spored with an apical ring, and 60–85 × 4.5–8 μm. Ascospores were ellipsoidal to fusiform, (0–)1-septate, constricted or not at septum, hyaline to light brown, smooth-walled, uniseriate, overlapping obliquely, and 10–15 × 4.5–5.3 μm.

**Colony characteristics:** On PDA, the colony was 20 mm in diam. after 1 week at 25 °C, the surface was cottony with dense, whitish aerial mycelium producing yellowish-brown pigments. On SNA, the colony was 26 mm in diam. after 1 week at 25 °C, the surface was velvet with sparse, whitish aerial mycelium. Conidiophores were verticillium-like, septate, and hyaline, with 1–2 whorls and a terminal whorl of 2–8 phialides, 30–120 μm long, and 2–3.5 μm wide at the base. Phialides were subulate, tapering toward the apex, 15–45 μm long, 1.5–2.5 μm wide at the base, and 0.2–0.3 μm wide at the apex. Macroconidia were ellipsoidal to fusiform or cylindrical, slightly curved, (0–)1(–3)-septate, smooth-walled, hyaline, and 10–28 × 3–5.5 μm.

**Notes:** Amongst the known species of the genus, *P. sinicus* is morphologically most similar to *P. macrosporus* Samuels in having superficial, solitary, non-stromatic, globose perithecia with warted surfaces; smooth-walled, 1-septate ascospores; and cylindrical, bicellular conidia [1]. However, *P. macrosporus* has a thicker perithecial wall (ca. 65 μm thick), clavate asci without apical rings, wider ascospores (5–7 μm wide), and longer conidia (33–47 μm long) [1]. In addition, there were 34 bp, 19 bp, 23 bp, 31 bp, and 23 bp divergences in the ITS, LSU, rpb1, rpb2, and tef1 regions between the ex-type cultures of the two species (CGMCC 3.24130 and CBS 423.88). Thus, both the morphological and the molecular evidence support their separation at the species level.

**Figure 6:**
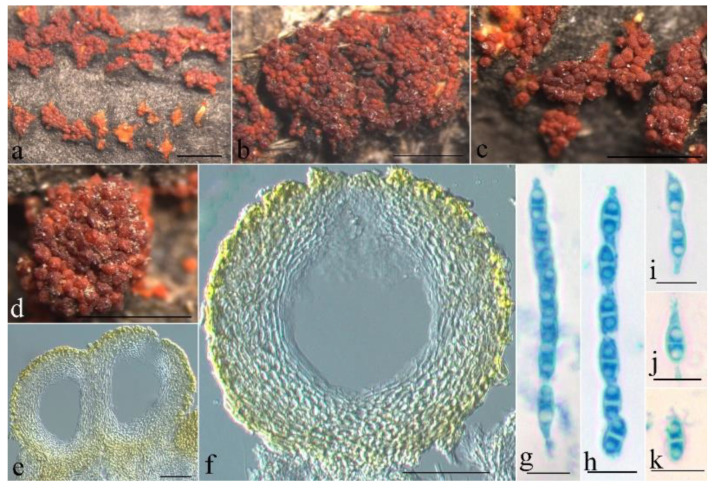
Macroscopic and microscopic morphology of *Pseudocosmospora beijingensis* (HMAS 290896). (**a**–**d**) Ascomata on natural substratum; (**e**,**f**) median section of perithecia in lactic acid; (**g**,**h**) asci with ascospores in lactophenol cotton blue; (**i**–**k**) ascospore in lactophenol cotton blue. Bars: (**a**–**d**) = 1 mm; (**e**,**f**) = 50 μm; (**g**–**k**) = 10 μm.

**Figure 7:**
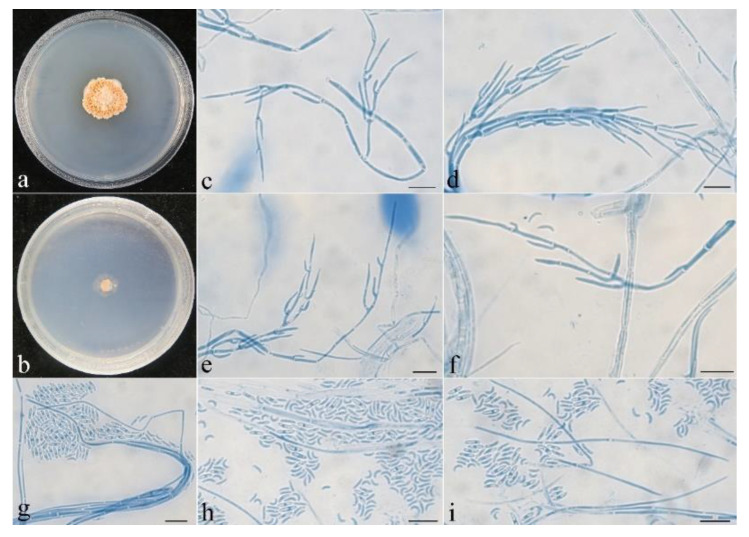
Colonial and microscopic morphology of *Pseudocosmospora beijingensis* (CGMCC 3.24131). (**a**) Colony after 1 week at 25 °C on PDA; (**b**) colony after 1 week at 25 °C on SNA; (**c**–**i**) conidiophores, phialides, and microconidia in lactophenol cotton blue. Bars: (**c**–**i**) = 10 μm.

***Pseudocosmospora beijingensis*** Z.Q. Zeng & W.Y. Zhuang, sp. nov. Figure 6 and Figure 7.

**Fungal Names:** FN571298.

**Etymology:** The epithet refers to the type locality of the fungus.

**Typification**: CHINA, Beijing, Beidagou forest, on rotten bark associated with other fungi, 10 August 2017, H.D. Zheng, X.C. Wang, Y.B. Zhang, C. Wang & P. Li 11339 (holotype HMAS 290896, ex-type strain CGMCC 3.24131).

**GenBank accession numbers:** ITS OP223438, LSU OP223434, tub2 OP272862.

Mycelium was not visible on the natural substratum. Perithecia were superficial and gregarious with a well-developed stroma, subglobose to globose, slightly roughened surface, laterally collapsed upon drying, orange-red to bright red, turning dark red in 3% KOH and light yellow in 100% LA, and 147–196 × 118–176 μm. Perithecial walls were two-layered, and 20–42 μm thick; the outer layer was of the textura globulosa to textura angularis, 15–25 μm thick, with cell 4–13 × 2.5–4.5 μm, and cell walls 1–1.2 μm thick; the inner layer was of the textura prismatica, 5–8 μm thick, with cell 6–10 × 2.5–3.5 μm, and cell walls 0.8–1 μm thick. Asci were unitunicate, cylindrical, with a simple apex, eight-spored, and 38–58 × 2.5–5 μm. Ascospores were ellipsoidal, 1-septate, not constricted at the septum, light yellow-brown, smooth-walled, uniseriate, and 8–10 × 2.5–4 μm.

**Colony characteristics:** On PDA, the colony was 25 mm in diam. after 1 week at 25 °C, surface crustose, producing yellowish-white pigments. On SNA, the colony was 15 mm in diam. after 1 week at 25 °C, surface velvet, with sparse, whitish aerial mycelium. Conidiophores were acremonium- to verticillium-like, septate, of indefinite length, and hyaline, with 1–2 whorls and a terminal whorl of 2–6 phialides. Phialides were subulate, tapering toward the apex, 10–55 μm long, 0.9–1.2 μm wide at the base, and 0.2–0.3 μm wide at the tip. Conidia were allantoid, curved, unicellular, smooth-walled, hyaline, and 2.6–4.5 × 0.9–1.8 μm.

**Notes**: Among the known species of *Pseudocosmospora*, *P. beijingensis* most resembles *P. curvispora* Z.Q. Zeng & W.Y. Zhuang in having subglobose to globose perithecia that are laterally collapsed upon drying; having asci without an apical ring; having ellipsoidal, 1-septate, smooth-walled, and light yellow-brown ascospores; and producing acremonium- to verticillium-like conidiophores, and allantoid, unicellular, curved conidia [8]. However, *P. curvispora* differs in clavate and somewhat longer asci (53–68 μm long) and narrower conidia (0.8–1.2 μm wide) [8]. Sequence comparisons revealed that there were 30 bp, 22 bp, and 90 bp divergences detected for the ITS, LSU, and tub2 regions. Obviously, they are not conspecific.

**Figure 8:**
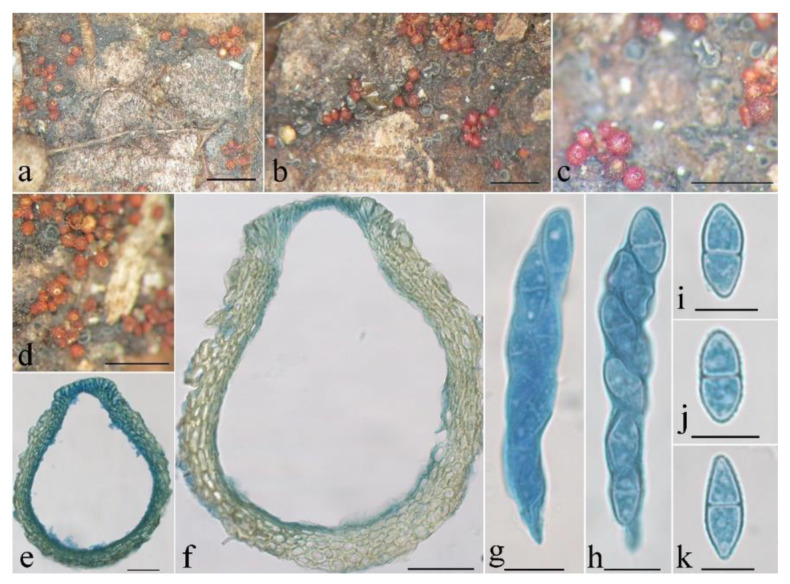
Macroscopic and microscopic morphology of *Thelonectria* *globulosa* (HMAS 255835). (**a**–**d**) Ascomata on natural substratum; (**e**,**f**) median section of perithecium in lactophenol cotton blue; (**g**,**h**) asci with ascospores in lactophenol cotton blue; (**i**–**k**) ascospore in lactophenol cotton blue. Bars: (**a**–**d**) = 1 mm; (**e**,**f**) = 50 μm; (**g**–**k**) = 10 μm.

**Figure 9:**
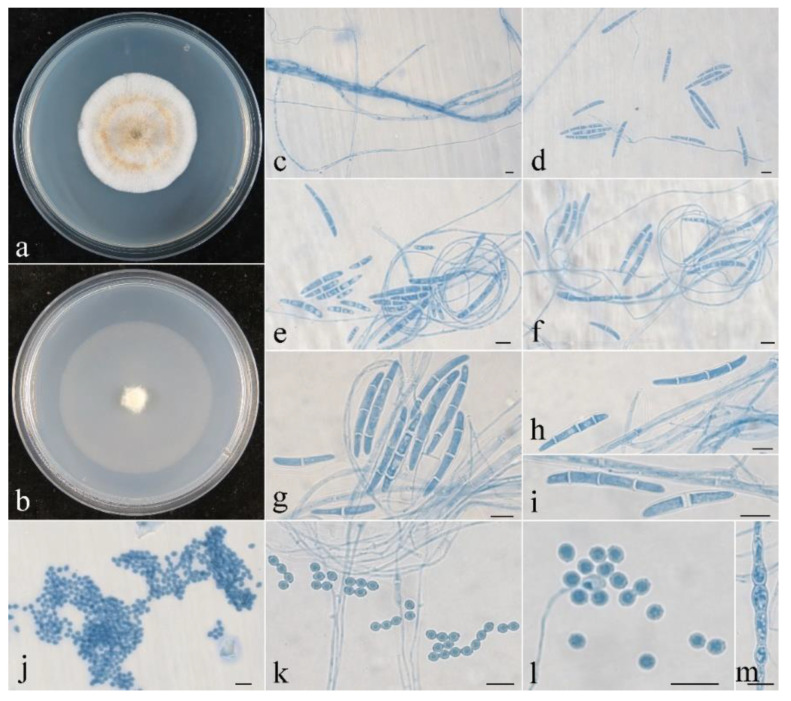
Colonial and microscopic morphology of *Thelonectria globulosa* (CGMCC 3.24132). (**a**) Colony after 2 week at 25 °C on PDA; (**b**) colony after 2 week at 25 °C on SNA; (**c**) conidiophores in lactophenol cotton blue; (**d**–**i**) conidiophores and macroconidia in lactophenol cotton blue; (**j**–**l**) microconidia in lactophenol cotton blue; (**m**) chlamydospores in lactophenol cotton blue. Bars: (**c**–**m**) = 10 μm.

***Thelonectria globulosa*** Z.Q. Zeng & W.Y. Zhuang, sp. nov. Figure 8 and Figure 9.

**Fungal Names:** FN571299.

**Etymology:** The epithet refers to the globose microconidia.

**Typification:** CHINA, Guangxi Zhuang Autonomous Region, Guilin City, Mao’er Mountain, on rotten roots, 5 December 2019, Z.Q. Zeng & H.D. Zheng 12434 (holotype HMAS 255835, ex-type strain CGMCC 3.24132).

**GenBank accession numbers:** act OP272865, ITS OP223436, LSU OP223432, rpb1 OP272867, rpb2 OP586760, tub2 OP586762.

Mycelium was not visible on the natural substratum. Perithecia were superficial, solitary to gregarious, with a basal or well-developed stroma, subglobose to globose, slightly roughened surface, with blunt papilla of 32–65 μm high and 52–75 μm wide at the base, did not collapse upon drying, orange-red to red, turning dark red in 3% KOH and light yellow in 100% LA, and 235–323 × 148–245 μm. Perithecial walls were two-layered, 20–40 μm thick; the outer layer was of the textura globulosa to textura angularis, 15–30 μm thick, with cell 5–15 × 4–12 μm, and cell walls 0.8–1 μm thick; the inner layer was of the textura prismatica, 5–10 μm thick, with cell 6–15 × 3–10 μm, and cell walls 1–1.2 μm thick. Asci were unitunicate, cylindrical to clavate, eight-spored, with a simple apex, and 53–75 × 8–13 μm. Ascospores were ellipsoidal to fusiform, 1-septate, constricted at the septum, hyaline, smooth to spinulose, uniseriate or irregular biseriate in asci, and 13–20 × 5.5–8 μm.

**Colony characteristics:** The colony on PDA was 22 mm in diam. after 1 week at 25 °C and had a cottony surface with dense, whitish aerial mycelium producing yellowish brown pigments. The colony on SNA was 35 mm in diam. after 1 week at 25 °C and had a cottony surface with sparse, whitish aerial mycelium. Conidiophores were mostly unbranched; rarely had simple branches; and were septate, hyaline, 25–89 μm long, and 1.5–2.5 μm wide at the base. Macroconidia were cylindrical to rod-shaped, slightly curved, 1–3(–4)-septate, smooth-walled, hyaline, and 20–58 × 3.2–5.8 μm. Microconidia were globose, smooth-walled, hyaline, and 3–4.5 μm in diam. Chlamydospores were globose to subglobose, and 4–10 × 3–8 μm.

**Notes:** Among the known species of *Thelonectria*, *T. globulosa* is distinct because of its globose microconidia. Morphologically, *T. globulosa* resembles *T. nodosa* C.G. Salgado & P. Chaverri in having solitary to gregarious, globose perithecia that do not collapse upon drying; cylindrical to clavate asci; ellipsoidal to fusiform ascospores; and cylindrical macroconidia. However, *T. nodosa* differs because of its larger asci (68–115 × 10–17 μm) with an apical ring, macroconidia possessing more septa (up to six septa), and lack of microconidia formation [12]. Moreover, there are 38 bp, 96 bp, 37 bp, and 65 bp divergences in the act, ITS, LSU, and rpb1 regions between the ex-type cultures of the two taxa (CGMCC 3.24132 and GJS 04155). Both the morphology and DNA sequence data distinguish them as different species.

**Figure 10:**
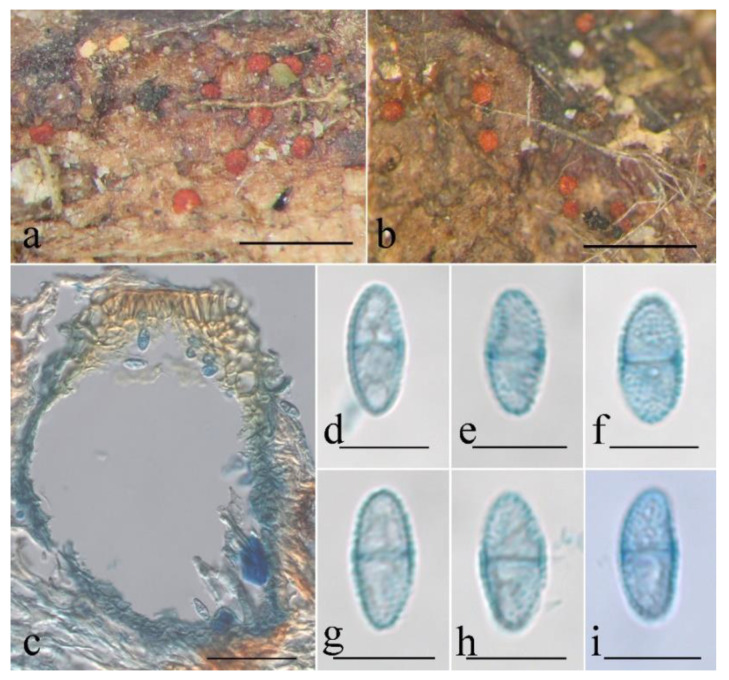
Macroscopic and microscopic morphology of *Thelonectria spinulospora* (HMAS 290897). (**a**,**b**) Ascomata on natural substratum; (**c**) median section of perithecium in lactophenol cotton blue; (**d**–**i**) ascospore in lactophenol cotton blue. Bars: (**a**,**b**) = 1 mm; (**c**) = 50 μm; (**d**–**i**) = 10 μm.

**Figure 11:**
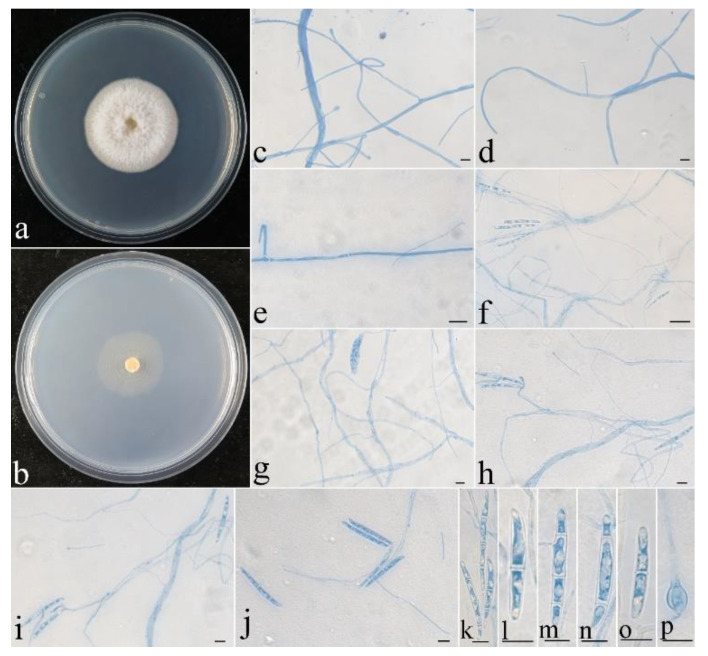
Colonial and microscopic morphology of *Thelonectria spinulospora* (CGMCC 3.24133). (**a**) Colony after 2 week at 25 °C on PDA; (**b**) colony after 2 week at 25 °C on SNA; (**c**–**e**) conidiophores in lactophenol cotton blue; (**f**–**j**) conidiophores and macroconidia in lactophenol cotton blue; (**k**–**o**) macroconidia in lactophenol cotton blue; (**p**) chlamydospore in lactophenol cotton blue. Bars: (**c**–**p**) = 10 μm.

***Thelonectria spinulospora*** Z.Q. Zeng & W.Y. Zhuang, sp. nov. Figure 10 and Figure 11.

**Fungal Names:** FN571300.

**Etymology:** The specific epithet refers to the spinulose ascospores.

**Typification:** CHINA, Guangxi Zhuang Autonomous Region, Guilin City, Mao’er Mountain, on rotten twigs, 7 December 2019, Z.Q. Zeng & H.D. Zheng 12499 (holotype HMAS 290897, ex-type strain CGMCC 3.24133).

**GenBank accession numbers:** act OP272866, ITS OP223437, LSU OP223433, rpb1 OP272868, rpb2 OP586761, tub2 OP586764.

Mycelium was not visible on the natural substratum. Perithecia were superficial, solitary, with a basal stroma, subglobose to broad-pyriform, surface slightly roughened, sometimes collapsed laterally upon drying, orange-red to red, turning dark red in 3% KOH and light yellow in 100% LA, and 123–195 × 143–212 μm. Perithecial walls were two-layered, 8–18 μm thick; the outer layer was of the textura globulosa to textura angularis, 5–13 μm thick, with cell 6–10 × 4–9 μm, and cell walls 1–1.2 μm thick; the inner layer was of the textura prismatica, 3–5 μm thick, with cell 2–8 × 3–10 μm, and cell walls 0.8–1 μm thick. Asci were not observed. Ascospores were ellipsoidal, 1-septate, not constricted or slightly constricted at the septum, hyaline, spinulose, and 12–18 × 5.6–8 μm.

**Colony characteristics:** On PDA, the colony was 40 mm in diam. after 2 weeks at 25 °C, the surface was cottony with dense, whitish aerial mycelium producing light yellow pigments. On SNA, the colony was 28 mm in diam. after 2 weeks at 25 °C, surface velvet, with sparse, whitish aerial mycelium. Conidiophores were acremonium-like, rarely with simple branches, septate, hyaline, and 45–102 × 2.2–4 μm. Macroconidia were cylindrical, slightly curved, (1–2–)3-septate, smooth-walled, hyaline, and 28–62 × 2.8–4.5 μm. Chlamydospores were globose to subglobose, smooth-walled, hyaline, and 6–8 μm in diam.

**Notes:** The morphological features, such as the superficial, globose to subglobose, broad-pyriform perithecia that do not collapse when dry; ellipsoidal, two-celled, and hyaline ascospores; and curved macroconidia with rounded ends, indicate the placement of *T. spinulospora* in *Thelonectria*, which was confirmed by sequence analyses of the act, ITS, LSU, rpb1, and tub2 regions (Figure 3). Amongst the known species of the genus, the new species is morphologically similar and phylogenetically related to *T. rubrococca* (Brayford & Samuels) C.G. Salgado & P. Chaverri in having solitary to gregarious, globose perithecia that do not collapse upon drying, ellipsoidal ascospores, and cylindrical macroconidia. However, the latter differs in its larger perithecia (200–450 μm in diam.), smaller ascospores (8–14.5 × 3.6–6.6 μm), and macroconidia with more septa (up to five septa) [38]. Sequence comparisons between the ex-type cultures of the two species revealed that 24 bp, 8 bp, 0 bp, 22 bp, and 28 divergences were detected for the act, ITS, LSU, rpb1, and tub2 regions. Both the morphology and DNA sequence data support their distinction at the species level.

## 4. Discussion

The genus *Penicillifer* is proposed as the preferable name over *Viridispora* [2], following the International Code of Nomenclature for algae, fungi, and plants [39]. Our analyses, inferred from sequences of ITS, LSU, rpb2, and tef1 and including the new taxon, revealed a tree topology (Figure 1) similar to that given by Lombard et al. [2]. The phylogenetic tree shows that *Penicillifer* species forms a well-supported monophyletic clade (MLBS/MPBP/BIPP/ = 96%/100%/100%) (Figure 1). *Penicillifer sinicus* is closely related to *P. macrosporus* (MLBS/MPBP/BIPP/ = 100%/99%/100%). The sequence comparisons revealed that there were 34 bp, 19 bp, 23 bp, 31 bp, and 23 bp differences detected for the ITS, LSU, rpb1, rpb2, and tef1 regions. Therefore, both the molecular and the morphological evidence supports the separation of the two fungi at a specific level. Among the known species of *Penicillifer*, *P. martinii* P. Wong, Y.P. Tan & R.G. Shivas is known solely by its sexual stage [5], and only asexual stages of *P. japonicus* Matsush. and *P. pulcher* have been discovered [4,40]; the rest species of the genus are holomorphic, including the newly added one.

Historically, nectriaceous species producing small, reddish, smooth, thin-walled perithecia were categorized as *Cosmospora* Rabenh. *sensu lato* [1]. The accumulated morphological and phylogenetic information indicated that the genus was not monophyletic [41,42]. Herrera et al. [6] established *Pseudocosmospora* to accommodate ten cosmospora-like fungi on *Eutypa* and *Eutypella* and with acremonium- to verticillium-like asexual stages. Since then, six additional taxa have joined the group [7,8,9]. The genus has become distributed worldwide and displays high species diversity in warm temperate and tropical regions [6]. Species of the genus have the following features in common: they are superficial perithecia, gregarious, KOH+, LA+, laterally collapsed upon drying, and usually less than 250 μm in height; and they have asci containing eight 1-septate ascospores, acremonium- to verticillium-like conidiophores, and non-septate conidia. *Pseudocosmospora beijingensis* fits well the generic concept. The multigene analyses indicated its distinctions from any other species of the genus (Figure 2).

Members of *Thelonectria* are often found on twigs and branches, trunks of recently killed or dying trees, and rotting roots; they occasionally cause small cankers and are mainly distributed in tropical, subtropical, and temperate regions [10,11,17]. Among the species of the genus, *T. coronata* (Penz. & Sacc.) P. Chaverri & C. Salgado, *T. discophora*, *T. lucida* (Höhn.) P. Chaverri & C. Salgado, and *T.* *veuillotiana* (Roum. & Sacc.) P. Chaverri & Salgado are cosmopolitan and are treated as species complexes [38,43,44]. Salgado-Salazar et al. [11,12,13] carried out a revisionary work on the above species complexes and described 30 cryptic species on the basis of genealogical concordance phylogenetic species recognition. Our phylogenetic results indicated that *T.* *globulosa* was associated with but clearly separated from members of the *T.* *veuillotiana* complex. *Thelonectria* *aurea*, known only by the asexual stage, can be easily distinguished in the absence of microconidia and chlamydospores in culture [17]. Moreover, there were 93 bp and 64 bp divergences in the ITS and tub2 regions between the ex-type culture of the two species.

There are 47 species currently known in this genus, of which 20 species have been reported in China [11,13,15]. Large-scale surveys of fungal resources in various regions with different climates, vegetation, geographic structures, and multiple niches will improve our understanding of the species diversity of nectriaceous fungi in the country.

## 5. Conclusions

The species diversity of the family *Nectriaceae* was investigated, and four novel taxa were discovered. With the joining of the new species, the phylogenetic relationships among species of these three genera were updated.

## Figures and Tables

**Table 1:** List of *Penicillifer* species, herbarium/strain numbers, and GenBank accession numbers of materials used in this study.

Species	Herbarium/Strain Numbers	GenBank Accession Numbers			
		ITS	LSU	rpb2	tef1
*Corallonectria jatrophae*	CBS 91396 ^T^	NR153873	KM231611	KM232298	KM231863
*Dematiocladium celtidis*	CBS 115994 ^T^	AY793430	AY793438	-	KM231864
*P. bipapillatus*	CBS 42088 ^T^	KM231740	KM231608	KM232295	KM231860
*P. diparietisporus*	CBS 37659 ^T^	NR154310	MH869437	KM232296	KM231861
*P. macrosporus*	CBS 42388 ^T^	MH862133	KM231607	KM232294	KM231859
*P. martinii*	BRIP 59225 ^T^	NR168155	NG068753	-	KJ869241
*P. pulcher*	CBS 56067 ^T^	NR154311	NG058093	KM232297	KM231862
*P. sinicus*	CGMCC 3.24130 ^T^	**OP223439 ^a^**	**OP223435**	**OP272863**	**OP272864**
*Stachybotrys chartarum*	CBS 12913	MH854622	MH866145	KM232434	KM231994

^T^ indicates the ex-type culture. ^a^ Numbers in bold indicate the newly provided sequences.

**Table 2:** List of *Pseudocosmospora* species, herbarium/strain numbers, and GenBank accession numbers of materials used in this study.

Species	Herbarium/Strain Number	GenBank Accession Numbers		
		ITS	LSU	tub2
*Corallomycetella repens*	AR 4547	JF832594	JF832679	JF832838
*Microcera larvarum*	AR 4580	KC291751	KC291759	KC291935
*P. beijingensis*	CGMCC 3.24131 ^T^	**OP223438 ^a^**	**OP223434**	**OP272862**
*P. curvispora*	CGMCC 3.20176 ^T^	MT592897	MT592879	MT606156
*P. eutypae*	CH 1101 ^T^	KC291735	KC291766	KC291925
	IMI 73016	KC291736	KC291786	-
	AR 4527	KC291720	KC291756	KC291909
*P. eutypellae*	AR 4562 ^T^	KC291721	KC291757	KC291912
	GJS 10248	KC291722	KC291772	KC291911
*P. henanensis*	HMAS 183528 ^T^	GU075856	GU075863	HM054103
*P. hypoxylicola*	cLL 19020 ^T^	MN886606	MN886608	-
*P. joca*	AR 4779 ^T^	KC291746	KC291762	KC291924
*P. metajoca*	AR 4576 ^T^	KC291745	KC291758	KC291923
*P. rogersonii*	GJS 9056 ^T^	KC291729	KC291780	KC291915
	GJS 10296	KC291727	KC291774	KC291917
	GJS 091384	KC291726	KC291770	KC291914
*P. shennongjiana*	CGMCC 3.20177 ^T^	MT592898	MT592880	MT606157
*P. vilior*	AR 4810 ^T^	KC291737	KC291763	KC291928
	AR 4771	KC291734	KC291761	KC291926
	PC 1246	KC291738	KC291791	KC291927

^T^ indicates the ex-type culture. ^a^ Numbers in bold indicate the newly provided sequences.

**Table 3:** List of *Thelonectria* species, herbarium/strain numbers, and GenBank accession numbers of materials used in this study.

Species	Herbarium/Strain Numbers	GenBank Accession Numbers				
		act	ITS	LSU	rpb1	tub2
*Cosmospora coccinea*	CBS 114050	GQ505967	FJ474072	GQ505990	GQ506020	DQ522501
*Nectria cinnabarina*	AR 4477/AR 4302	HM484627	HM484548	HM484562	M484577	HM484820
*T. acrotyla*	GJS 90171	JQ365047	JQ403329	JQ403368	JQ403407	JQ394720
*T. amamiensis*	MAFF 239819	JQ365054	JQ403337	JQ403375	KJ022408	JQ394727
*T. chlamydospora*	ST 121 ^T^	LC519560	LC509450	LC509452	-	-
*T. globulosa*	CGMCC 3.24132 ^T^	**OP272865 ^a^**	**OP223436**	**OP223432**	**OP272867**	**OP586762**
*T. gongylodes*	GJS 04171 ^T^	JQ365038	JQ4033	JQ403358	JQ403395	JQ394710
*T. nodosa*	GJS 04155 ^T^	JQ365037	JQ403317	JQ403357	JQ403394	-
*T. olida*	CBS 21567 ^T^	HM352884	KJ021982	KJ022058	HM364334	KM232024
*T. rubrococca*	IMI 324475 ^T^	KJ022275	KJ022008	KJ022061	KJ022439	KJ022329
*T. spinulospora*	CGMCC 3.24133 ^T^	**OP272866**	**OP223437**	**OP223433**	**OP272868**	**OP586764**
*T. torulosa*	AR 4768A	JQ365031	JQ403310	JQ403350	JQ403386	JQ394702
*T. trachosa*	GJS 9245 ^T^	KF569832	KF569842	KF569851	KF569879	KF569869
*T. truncata*	GJS 04357 ^T^	JQ365039	JQ403319	JQ403359	JQ403396	KJ022324
*T. veuillotiana*	AR 1751	KJ022273	JQ403305	JQ403345	JQ403382	JQ394698

^T^ indicates the ex-type culture. ^a^ Numbers in bold indicate the newly provided sequences.

## Data Availability

Names of the new species were formally registered in the database Fungal Names (https://nmdc.cn/fungalnames (accessed on 22 August 2022)). Specimens were deposited in the Herbarium Mycologicum Academiae Sinicae (HMAS). The newly generated sequences were deposited in GenBank (https://www.ncbi.nlm.nih.gov/genbank (accessed on 5 October 2022)).
